# Integrating tuberculosis care cascade activities into medical education and training: experiences from a pilot intervention from rural Maharashtra, India

**DOI:** 10.3389/fpubh.2025.1497474

**Published:** 2025-05-21

**Authors:** Swathi Krishna Njarekkattuvalappil, Saibal Adhya, Sanjivani Vishwanath Patil, Sanjay Sonyabapu Darade

**Affiliations:** ^1^Department of Community Medicine, Bharati Vidyapeeth Medical College, Pune, India; ^2^District TB Centre, Maharashtra State Health Services, Pune, India

**Keywords:** family adoption programme, active case finding, tuberculosis care cascade, medical education, screening

## Abstract

India has the highest burden of tuberculosis (TB) in the world. The mandatory Family Adoption Programme (FAP) visits to adopted villages by medical college teams is an excellent opportunity to do the ideal “community screening” for TB in a door-to-door manner. We nested an active case finding activity for TB in the FAP visits by MBBS students in rural Pune, Maharashtra and the learnings and recommendations from this pilot intervention are detailed here. It is a sustainable and replicable activity for MBBS students and a great opportunity to collaborate with the national health programme.

## Family adoption programme (FAP) and its potential

Family Adoption Programme (FAP) is a mandatory activity under the leadership of Community Medicine departments, as per the Competency Based Medical Education (CBME) for undergraduates by National Medical Commission (NMC) to orient medical students toward felt needs of the community by working amidst them first-hand, especially in rural India, and hence bridge the increasing urban-rural health divide in our country (1). Medical colleges are also advanced tertiary healthcare institutes serving medically complicated, critically ill patients, and places where clinical training happens. A doctor-in-the-making should be trained adequately to become astute enough to identify common healthcare problems of the community and be able to provide comprehensive primary healthcare in resource-limited settings, which is the reality in majority of India. Similar to the erstwhile Re-Orientation of Medical Education (ROME) postings, the current FAP posting is an effort toward shaping a community health provider who can deliver quality healthcare across settings (2, 3). Hence a close collaboration with the healthcare service delivery system and medical education system is required.

**Figure 1:**
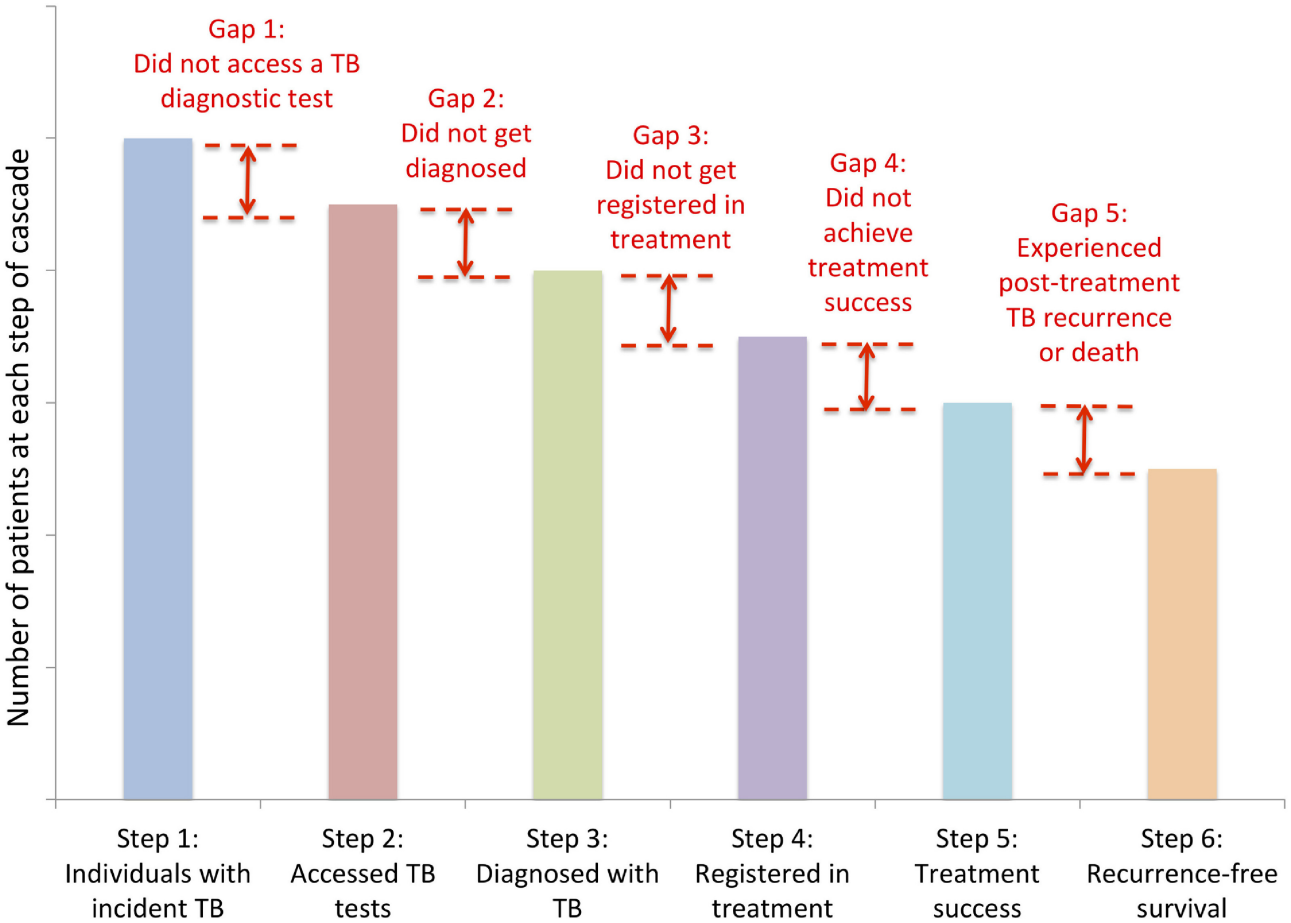
Care cascade for tuberculosis with potential gaps. Source: Subbaraman et al. (7). Image reused with Elsevier license permission no: 5820720225690.

India is the leader in global tuberculosis (TB) burden and has 27% of the reported persons with TB (PwTB) (4). The state of Maharashtra is one among the leading contributors to the country's TB burden, it notified about 2.34 lakh cases in 2022, about 10% of total notified cases in India (5). Almost 64% of the symptomatic population in India do not seek healthcare, mainly due to ignoring of symptoms and lack of awareness. Almost half the patients presenting with classical TB symptoms are missed from being diagnosed also (6). One of the commonest gaps in TB care cascade is the delay in identifying a person with presumptive TB and doing a test for diagnosis (Figure 1). Early diagnosis and integration into TB care cascade require heightened level of awareness and action both from the community and healthcare providers (7).

Active Case Finding (ACF) is one of the strategies used by the National Tuberculosis Elimination Programme (NTEP) to detect missing PwTB. As per WHO guidelines, this activity is to be done in high-risk populations, but can also be done among general population with an estimated TB prevalence of 0.5% (8). NTEP has been implementing ACF among high risk populations in all districts since 2017 (9). The usual algorithm used in the ACF activity of NTEP is a combination of WHO recommended 4-symptom screen, chest X-ray and WHO-recommended rapid molecular diagnostics (CBNAAT or Truenat). Though it is ideal and useful to do community-wide screening in high burden settings, it is very resource-intensive in programme mode (10).

## Pilot intervention in rural Pune district

While the routine ACF rounds of NTEP cover the high-risk populations, the FAP visits to adopted villages by medical college teams is an excellent opportunity to do the ideal “community screening” in a door-to-door manner. Each MBBS student is allotted 4–5 households in the respective adopted villages, to be followed up for next 3 years in terms of overall health and development. Hence the department of Community Medicine of Bharati Vidyapeeth Medical College, Pune initiated the nesting of an ACF activity for TB within the routine FAP household visits by MBBS students. The adopted villages for FAP of the department are in the Mulshi taluka of rural Pune, which belongs to the Paud TB unit (TU) of the district NTEP. Paud TU notified 1,057 cases of TB (13% of total notified cases from Pune Rural) in the year 2022, second highest among the 14 TUs constituting Pune rural district (11). After detailed discussions and meetings with the District NTEP to collaboratively execute this project, this intervention was done in Bhukum and Ambarwet *grampanchayats* of Paud TU from March to June 2024. The WHO recommended 4-symptom screen used by NTEP was used as screening tool. All individuals present in the household during the visit, including children were screened.

The questionnaire to be used for screening was finalized by the District NTEP (author SSD) along with the faculty of department of Community Medicine (SKN, SA). This was in line with the questionnaire used for ACF by District NTEP (Table 1). Then, a two-stage training was conducted, first for all the team members like faculty, medical social workers (MSWs), post graduate residents and MBBS interns, prior to the field visits. The second stage of questionnaire training was given by SKN and SVP to five batches of 30–35 MBBS students each, prior to their visit to households. At the field, each student batch was divided into 5 groups with an MSW as in-charge, along with a post graduate resident and two MBBS interns to guide the process.

**Table 1:** Questionnaire for ACF for TB among FAP households.

**Sl No**.	**Question to be asked to each family member**	**Instruction**
1	Do you have any of the following symptoms? •Cough for more than 14 days •Fever for more than 14 days/fever with night sweats •Weight loss •Blood in sputum	If answer to **any one** of these is “yes,” then that person is identified as a “presumptive TB” person.
2	Is/are there any member(s) in your family who is currently taking TB treatment?	Any “yes” to be reported as a person with active TB
3	Was there any member in your family who was diagnosed with TB/took treatment for TB in the past 5 years?	Any “yes” to be reported as a source of contact

The students reported the numbers obtained to the respective MSW-in-charge of their group. All numbers were captured on hardcopy forms. Any symptomatic individual identified was examined by the postgraduate resident and discussed with faculty on field about next steps. Further follow up of identified presumptive TB individuals were done in coordination with the local NTEP staff. They were connected to local Accredited Social Health Activist (ASHA) or Senior Treatment Supervisor (STS) for counseling and testing. In addition to this, students provided health education to the community on TB in the form of street plays, talks etc. The reports of the activity were later collated by the investigators. Five hundred and fifty-nine individuals were screened in the activity from two villages, presumptive TB identified were connected to NTEP for testing (Table 2). The two presumptive TB individuals identified underwent sputum smear microscopy and were found to be negative for *Mycobacterium tuberculosis*. One hundred fifty undergraduate students, 5 postgraduate students, 25 MBBS interns and 6 medical social workers were trained in the process. So far, roughly 3% of the total population of the taluka has been screened. But since this is now integrated as a routine activity in the FAP visits, we expect to cover more population in subsequent visits occurring every 6 months in a different village of the taluka (12). Currently, the activity is ongoing in Shindewadi, another village of the same taluka.

**Table 2:** Cumulative numbers of ACF activity in two villages of Paud TU.

**Sl No**.	**Indicator**	**Bhukum village**	**Ambarwet village**	**Total**
1	Number of individuals screened for TB	440	119	559
2	Number of presumptive TB persons identified	1	1	2
3	Number of active TB persons identified	0	0	0
4	Number of household contacts/close contacts identified	2	2	4

## Discussion

Medical college training in the past, used to focus on nurturing clinical knowledge alone. But with the advent of CBME, this is changing. A clinician faces a lot of dilemmas while translating their knowledge into practice. This constraint could be addressed only if a medical student is trained well-connected to the real-world settings, right from the beginning. Awareness about health policies and programmes of the country is essential for practicing clinicians for imparting quality patient care. Tuberculosis is one of the national health priorities, where the government of India is investing a lot of resources on, along with international support (5). We have a well-functioning national health programme, financial support provisions and highest level of political commitment for ending TB. Despite these, India accounts for 18% of the global gap between estimated TB incidence and the reported number of people newly diagnosed with TB (4). Standardized patient studies conducted in Mumbai and Patna have shown that MBBS practitioners could correctly presume and manage a classical symptomatic pulmonary TB presentation in only 54% instances (13). This shows the dire need for proper training from early days itself.

The medical colleges come under the Directorate of Medical Education and also with the advent of large number of private and deemed institutions, the connection with the public healthcare delivery system and execution of national health programmes, which comes under the Directorate of Health Services, is becoming much fainter. Mutual efforts between the teaching institutions and healthcare delivery system must exist to facilitate learning relevant to community needs, as demonstrated by this pilot project. One of the best health systems in the world is that of Cuba, where 75–80% of medical training takes place in community primary care facilities with an accredited polyclinic acting as the central teaching unit. The specialists in Comprehensive General Medicine, called doctor-tutors, perform their basic public health functions within the national health service—teaching, medical practice, research and administration—whilst being responsible for groups of students (14). This model empowers the students to work within varied settings after completing their graduation, and not be weighed down by harsh realities of the field, as seen in the study by Ramani et al. (15).

Few learnings and recommendations from the execution of this pilot project in TB screening are as follows:

- Conceptual learning than yield of screening: the focus of this activity was to help students learn basic concepts like active case finding, presumptive tuberculosis, active tuberculosis and 4-symptom screening for TB, rather than the yield of screening activity. It also helped them in understanding importance of precise and relevant questions in history taking.
- In-line with CBME curriculum: integrated teaching practiced in CBME enables students to understand disease conditions and other topics in continuum and in totality. Screening activity done by the student in the community for an infectious disease like tuberculosis, satisfies the highest level of competency- “perform”- on the Miller's pyramid (16).
- Public-private partnership model: even though NTEP has been utilizing the skilled human resources of medical colleges for sub-national certification of TB elimination activity, our intervention is yet another excellent example of public-private partnership in TB care and prevention, by demonstrating efficient utilization of available human resources to carry out important activities like disease screening and harnessing the potential of hitherto underutilized pool of public health expertise in medical college faculty.
- Making TB screening a routine practice: during these times when our country is working hard toward attaining end TB targets, it is imperative that the budding doctors be trained in less resource intensive practices like 4-symptom screening, which could be routinized and easily implemented at all levels.
- Sustainable screening model: since the adopted villages are allotted on a rotation basis to each yearly MBBS batch, a constant cycle of screening and follow-up is possible in the specific geographic area. We already have evidence from Vietnam that a continued active screening for TB among general population in high burden settings can reduce the incidence over years (10).
- Advocacy among community: the screening activity was also a great method and opportunity to impart health education on TB to the rural community. The probing into symptoms helped create an awareness about symptoms suggestive of TB and importance of getting timely testing. Similar initiatives were taken up and implemented effectively by community members, like in Tanzania, where door-to-door screening was carried out by community volunteers (17).
- Integration potential with existing initiatives: the ongoing community engagement initiative for ending TB, the TB *Mukt Panchayat* initiative (TBMPI), is a potential venture with which medical colleges can collaborate and provide assistance. Given the fact that FAP happens in *gram panchayat* areas, a tie-up between medical colleges and respective *panchayats* could be mutually beneficial in carrying out the stipulated activities of TBMPI, including screening and advocacy.
- Replicable model for nesting other national health programmes: community based screening for TB by medical students is already demonstrated to be feasible, provided the programme strengthens the testing and treatment capacities of peripheral health centres (18). Similar activities related to other relevant national health programmes could be nested in FAP in medical colleges across the country. For instance, screening for communicable diseases like leprosy and non-communicable diseases like diabetes, hypertension, mental health issues etc. could be carried out by using simple field questionnaires and point-of-care tests.
- Donor funded projects to explore collaborative activities: donor-funded projects in health programmes rarely explore the potential of medical teaching institutions as partners on ground. The Community Medicine departments could be more extensively involved in field activities of these projects, providing opportunities for postgraduate residents and faculties to supplement the same. For example, the United States Agency for International Development (USAID) funded Tuberculosis Implementation Framework Agreement (TIFA) Project has leveraging and advocating for additional domestic resources for TB from the public and private sectors as one of its objectives. The grants from this project to support partners to test new interventions and demonstrate models for service provision to better meet the needs could include medical colleges too. Also, the Global Fund financed Project Axshya worked on the principle of community participation in TB care and control. One of the major activities in the project was active case finding for TB using trained community volunteers. This could have been done involving medical teaching institutes also.

A limitation to this intervention is that we relied only on the WHO recommended 4-symptom screening tool for TB screening, which has a sensitivity of only 71% (8). Nevertheless, our intention was to pilot a model which requires minimal resources, facilitate conceptual learning for students and help in generating awareness in community, all of which were fulfilled by this tool. Another challenge during the intervention was the reliance on reported symptoms by the participants to identify them as presumptive TB. As shown by several studies in high burden settings, TB is a highly stigmatized disease and people are generally hesitant to share any symptoms to healthcare workers fearing discrimination, hence the results of the enquiry about 4-symptoms is definitely an underestimate of actual truth (19, 20).

## Conclusions

FAP in Community Medicine syllabus for MBBS students in India is an excellent platform for incorporating TB care cascade activities and training future doctors by providing hands-on learning opportunities via skill-based learning.

## Data Availability

The original contributions presented in the study are included in the article/supplementary material, further inquiries can be directed to the corresponding author.
